# Citelli’s Abscess Following Otitis Media: A Case Report

**Published:** 2017-05

**Authors:** Anjan-kumar Sahoo, Chappity Preetam, Dillip- kumar Samal, Sourav Sarkar

**Affiliations:** 1 *Department of ENT and Head and Neck Surgery, All India Institute of Medical Science, Bhubaneswar, Odisha, India.*

**Keywords:** Citelli's abscess, Extratemporal complication of otitis media, Mastoidectomy

## Abstract

**Introduction:**

Citelli’s abscess is an extratemporal complication of otitis media. It occurs when pus from the mastoid tip trickles down along the posterior belly of the digastric muscle to the occipital and cervical region. It is a very unusual presenting complication of chronic otitis media with no available data in the until now.

**Case Report:**

A 10-year-old female was presented to our outpatient department with a 1 month history of hi-grade fever and headache and pain around the left half of the face. During physical examination a huge swelling present in the left temporal and occipital region was observed. The swelling crossed the midline, was tender to touch, and was fluctuant. During otological examination left sided chronic suppurative otitis media, of the attico-antral type with cholesteatoma, and a profuse foul smelling purulent discharge was observed. After complete investigation, drainage of the patient’s abscess was performed under general anesthesia. A postaural incision was administered and around 500 ml of pus drained out. Immediately after the operation, the patient showed signs of recovery. After 3 weeks of parenteral antibiotic therapy, the primary focus was debrided by performing left modified radical mastoidectomy.

**Conclusion:**

Citelli's abscess is a rare complication of otitis media. Urgent radiology, followed by drainage of pus is performed to reduce pain and further progression of the infective process. The primary ear pathology is managed surgically after adequate treatment with intra venous antibiotics.

## Introduction

The incidence of otitis media induced complications has been significantly reduced with the availability of good quality antibiotics; however, in developing countries, this complication is still occasionally encountered (1). Otitis media can be either mucosal or squamosal type, with complications being more common in the squamosal type. A common complication of OM is acute mastoiditis which can lead to a variety of abscesses (2). Citelli’s abscess is an extratemporal complication of otitis media. It occurs when pus from the mastoid tip trickles down along the posterior belly of the digastric muscle to the occipital and cervical region. It is a very unusual presenting complication of chronic otitis media with no available data in recent literature. In this paper, we report a very interesting and rare case of Citelli’s abscess that presented to us as an occipital swelling.

## Case Report

**Fig1:**
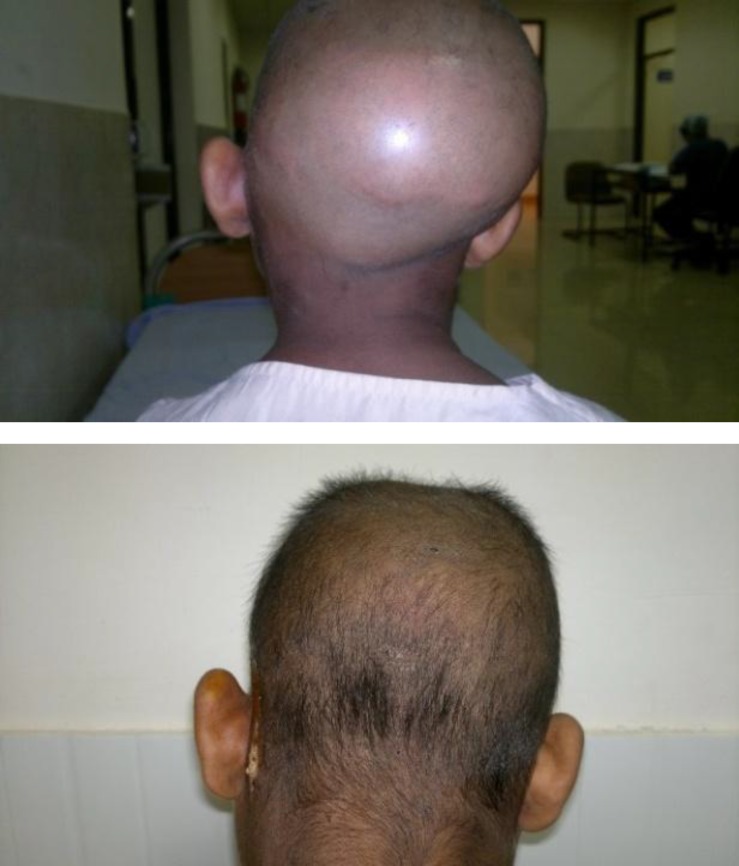
Pre and post-operative pictures of the patient

A 10-year-old Indian girl presented to our outpatient department with a 15-day history of hi-grade fever and headache and pain around the left half of face. Apparently, she had a history of left ear discharge since the age of two, which was being treated conservatively. During examination, the patient was febrile and toxic. During physical examination, a huge swelling in the left temporal and occipital region, which was crossing the midline and was tender and fluctuant, was observed (Fig. 1). 

**Fig 2:**
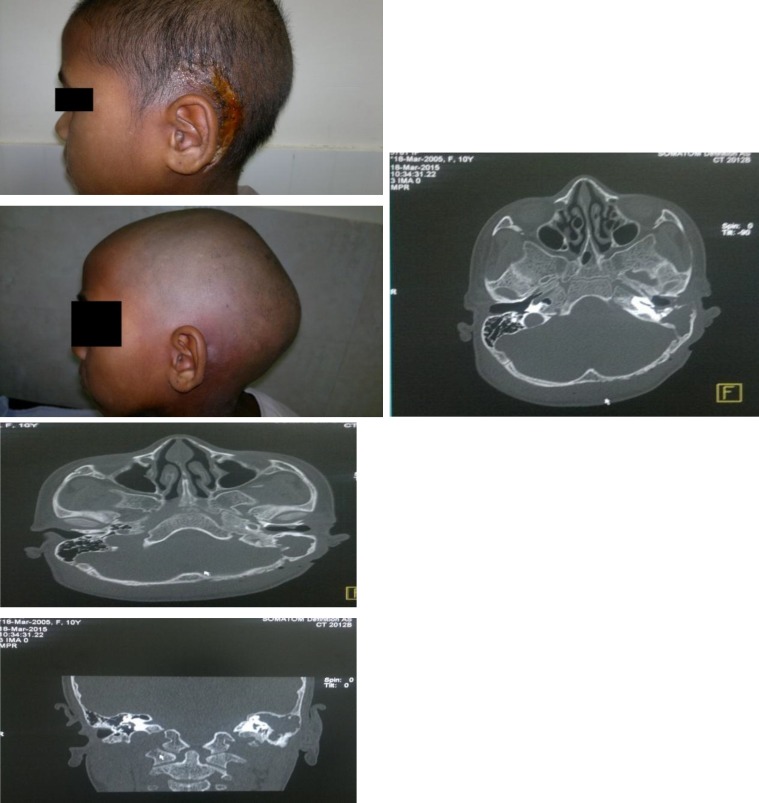
High resolution computed tomography of the temporal bone of the patient showing a soft tissue mass in the mastoid antrum, middle ear, left temporal and occipital area with erosion of the mastoid tip

During examination of the left ear, a cholesteatoma with a foul smelling discharge was observed. The right ear was normal. No signs were suggestive of meningeal irritation. Fundus examination was normal. Hematological investigations showed that the patient had a hemoglobin count of 8.2 g/dl, a total leukocyte count of 22300/mm3 with neutrophilic leukocytosis (88% neutrophils on the differential count), and a normal platelet count. The erythrocyte sedimentation rate was 48 mm in the first hour. The renal function tests and serum electrolytes were within normal limits. Audiometry revealed severe conductive hearing loss in the left ear with normal hearing levels in the right ear. A high resolution computed tomogram (HRCT) of the temporal bone (Fig. 2) revealed a soft tissue mass in the mastoid antrum and middle ear with erosion of the mastoid tip.

*Treatment*

After complete evaluation, the drainage of the patient’s abscess was performed under general anesthesia. A postaural incision was administered and around 300-400 ml of pus was drained out. Post-drainage, the patient’s general condition improved significantly. The patient became afebrile and counts decreased progressively. After 3 weeks of parenteral antibiotic therapy, the primary focus was addressed by performing a left modified radical mastoidectomy with middle temporal artery based flap obliteration of cavity.

## Discussion

Complications associated with OM are not very rare in the developing countries. Though the incidence had decreased, it is still a common menace. Complications of otitis media occur when the infective process spreads beyond the confines of the middle ear. The various pathways for the spread of infection are by direct extension through bone, by cholesteatoma or chronic osteomyelitis, through the spread by thrombosis of small venules from the dura, transverse sinus and beyond; and through preformed pathways, such as labyrinth, the endolymphatic channels and developmental or traumatic bony defects. Though OM is histologically benign, the invasive properties of cholesteatoma are caused by the enzymatic destruction of bone which leads to the various complication like mastoid abscess, brain abscess and meningitis. Mastoid abscess is the most common extracranial and overall the most common complication of CSOM. Further spread of the disease may lead to Bezold’s abscess, Citelli’s abscess, Lucs abscess and Zygomatic abscess (3). Citelli’s abscess occurs either because of direct extension of pus from the mastoid tip area through the facial or subcutaneous plane or through the mastoid emissary vein (4). Radiology is mandatory prior to incision and drainage, to rule out other associated complications. Drainage of pus relieves pain and improves patient’s condition. More importantly it inhibits further spread to the intracranial cavity. The definitive surgery is planned after the completion of 2-3 week course of antibiotics. Modified radical mastoidectomy is performed in cases where hearing is preserved. Obliteration of cavity is performed primarily to prevent any further spread of infection. It also aids in early healing and avoids recurrent discharge. Vascularized flaps are preferred for obliteration, as they do not necrose and prevent infections effectively.

## Conclusion

Citteli's abscess is a rare complication of COM. Urgent radiology, followed by drainage of pus is performed to reduce pain and further progression of the infective process. Primary ear pathology is managed surgically after adequate treatment with intra venous antibiotics. The obliterated cavities need to be followed up regularly with radiology to identify any early recurrence of the disease process.
